# Towards Strain-Level Complexity: Sequencing Depth Required for Comprehensive Single-Nucleotide Polymorphism Analysis of the Human Gut Microbiome

**DOI:** 10.3389/fmicb.2022.828254

**Published:** 2022-05-05

**Authors:** Pu Liu, Shuofeng Hu, Zhen He, Chao Feng, Guohua Dong, Sijing An, Runyan Liu, Fang Xu, Yaowen Chen, Xiaomin Ying

**Affiliations:** ^1^Center for Computational Biology, Beijing Institute of Basic Medical Sciences, Beijing, China; ^2^Yongkang First People’s Hospital, Yongkang, China

**Keywords:** gut microbiota, strain level, SNP, metagenomic sequencing, sequencing depth

## Abstract

Intestinal bacteria strains play crucial roles in maintaining host health. Researchers have increasingly recognized the importance of strain-level analysis in metagenomic studies. Many analysis tools and several cutting-edge sequencing techniques like single cell sequencing have been proposed to decipher strains in metagenomes. However, strain-level complexity is far from being well characterized up to date. As the indicator of strain-level complexity, metagenomic single-nucleotide polymorphisms (SNPs) have been utilized to disentangle conspecific strains. Lots of SNP-based tools have been developed to identify strains in metagenomes. However, the sufficient sequencing depth for SNP and strain-level analysis remains unclear. We conducted ultra-deep sequencing of the human gut microbiome and constructed an unbiased framework to perform reliable SNP analysis. SNP profiles of the human gut metagenome by ultra-deep sequencing were obtained. SNPs identified from conventional and ultra-deep sequencing data were thoroughly compared and the relationship between SNP identification and sequencing depth were investigated. The results show that the commonly used shallow-depth sequencing is incapable to support a systematic metagenomic SNP discovery. In contrast, ultra-deep sequencing could detect more functionally important SNPs, which leads to reliable downstream analyses and novel discoveries. We also constructed a machine learning model to provide guidance for researchers to determine the optimal sequencing depth for their projects (SNPsnp, https://github.com/labomics/SNPsnp). To conclude, the SNP profiles based on ultra-deep sequencing data extend current knowledge on metagenomics and highlights the importance of evaluating sequencing depth before starting SNP analysis. This study provides new ideas and references for future strain-level investigations.

## Introduction

In recent years, metagenomic studies have deepened our understanding of the relationships between the human gut microbes and diseases. Intestinal microbes have been reported to be closely related to digestive tract diseases (Sun et al., 2017), cardiovascular diseases (Li et al., 2017), metabolic diseases (Maruvada et al., 2017), immune diseases (Fujimura and Lynch, 2015), neurological diseases (Qian et al., 2020) and even tumors (Jin et al., 2019). In metagenomic studies, strain-level analysis has attracted more and more attention. Different strains within a species may exhibit high variability in pathogenicity, antibiotic resistance and interactions with the host in response to the environmental stress (Anyansi et al., 2020), despite that they are similar in genomic content (Van Rossum et al., 2020). For example, *Escherichia coli* O157:H7 has caused water-borne and food-borne disease outbreaks in many countries (Manning et al., 2008), while *Escherichia coli* sequence type 95 strains are antibiotic resistant (Stephens et al., 2017). Strain-level variation in the microbiome of diabetic wounds has also been proved to be associated with clinical outcomes and therapeutic efficacy (Kalan et al., 2019). Therefore, in metagenomic studies, it is far from enough to conduct a coarse-grained research at species level, especially in studies on infectious disease (Yan et al., 2020).

Metagenomic single-nucleotide polymorphisms (SNPs) have been utilized to disentangle conspecific strains to facilitate strain-level investigations such as strain identification and tracking (Costea et al., 2017; Anyansi et al., 2020). As the indicator of strain-level complexity, several SNP-based tools have been developed to identify strains in metagenomes. For example, StrainFinder uses SNP-based methods to calculate abundances for all the genomes in the reference database (Smillie et al., 2018). StrainEST is based on co-occurring SNPs within samples to predict abundances of strains (Albanese and Donati, 2017). By using different tools, substantial associations between metagenomic SNPs and host phenotypes have been uncovered. Schloissnig et al. first described the genomic variation landscape of the healthy human gut microbiome and found that subjects exhibited more individuality and temporal stability of metagenomic SNP variation patterns than composition changes of their gut microbiota (Schloissnig et al., 2012). Chen et al. found that there is a close relationship between type 2 diabetes and human intestinal metagenomic SNPs (Chen et al., 2017). Li et al. found that gut microbial SNPs induced by high-fiber diet dominate nutrition metabolism and environmental adaption of *Faecalibacterium prausnitzii* in obese children (Li et al., 2021). Besides, Chen et al. found that the SNPs and structural variations (SV) of multiple bacterial species in the human intestinal tracts are stable to build microbial fingerprints for people (Chen L. et al., 2021).

Although next generation sequencing data greatly accelerates metagenomic discoveries, few works have studied the impact of sequencing depth on the identified SNP profile and strain-level composition of the gut microbial community. This cannot be ignored because sequencing depth directly impacts the result of read assignments and SNP calling. Two sequencing strategies, ultra-deep sequencing and shallow sequencing, can be adopted in metagenomic SNP studies. Ultra-deep sequencing represents the sequencing strategy to obtain whole genome sequences at very high depths, whereas shallow sequencing represents the strategy to obtain genomes at relatively lower depths. A shallow sequencing depth may lead to miss of important information and result in biased results. The sufficient sequencing depth for strain-level analysis remains unclear.

Hence, we explored the human intestinal metagenomic SNP profiles by using ultra-deep sequencing. We investigated the impact of sequencing depth on identification of metagenomic SNPs and downstream strain-level analysis. We hope to provide new ideas and references for future metagenomic SNP researches.

## Materials and Methods

### Description of the Dataset

In total, three ultradeep sequenced fecal samples were utilized in this study. Of which, one from a healthy Chinese person named D1 was sequenced by our group with a sequencing size of 437 GB (Gigabases), and the other two samples that named D2 (BioSample: SAMEA5669780) and D3 (BioSample: SAMEA5669781) from the United States were downloaded from the European Nucleotide Archive (ENA) with the accession ID PRJEB24152 (Hillmann et al., 2018). The sequencing sizes of D2 and D3 are 786 GB and 754 GB, respectively.

### Metagenome DNA Extraction and Shotgun Sequencing

Microbial genomic DNA from stool of the Chinese sample was extracted using Tiangen Fecal Genomic DNA Extraction Kit (Tiangen Biochemical Technology Co., Ltd.) in line with the standard procedure, and was stored at −80°C. The purity and concentration of DNA was measured with agarose gel electrophoresis and an optical density (OD) analysis with NanoDrop spectrophotometer (Thermo Fisher Scientific, United States). The DNA concentrations were also measured with Qubit™ 4.0 Fluorometer (Thermo Fisher Scientific, United States). Shotgun DNA sequencing was performed on the Illumina NovaSeq 6000.

### Quality Control of Reads

FastQC (Andrews, 2010)^1^ was used to check the quality of raw data. We then implemented a customized quality control strategy by combining Trimmomatic (parameters: -phred33 ILLUMINACLIP:adaptors.fa:1:0:7 TRAILING:20 SLIDING-WINDOW:5:10 MINLEN:45 AVGQUAL:20) (Bolger et al., 2014) with in-house scripts.^2^ Briefly, the quality control was carried out with the following criteria: (1) Adaptors are removed; (2) Bases with qualities smaller than 20 are trimmed from 3’ end; (3) In a 5mer sliding window of a read, if the average quality within the window falls below 10, the read is clipped; (4) Reads containing less than 45 bases are dropped; (5) Reads are also dropped if the average quality is blow 20; (6) For each site of all reads in each sample, a mean number of base calls (*f*) and a standard deviation (SD) were calculated. Sites whose bases call number deviated from its 2 standard deviations were cut. Steps (1) – (5) are performed by Trimmomatic and step (6) is performed by our custom script.

### Downsampling

Each of the three samples was randomly down-sampled to 1,000, 10,000, 1,00,000, 1 million, 10 million, 20 million, 30 million, 40 million, 50 million, 60 million, 80 million, 100 million, 200 million, 300 million, 400 million, 500 million, 1 billion and 2 billion sequencing reads as subsamples using BBMap (Bushnell, 2014), respectively.

### Computation of Relative Strain Abundance

If a strain *s* with genome length *l*_*s*_ has *n*_*s*_ uniquely mapped reads assigned to its genome, we defined the relative abundance of strain *r*_*s*_ as follows:


$${\text{r}}_{s}=\frac{{\text{n}}_{s}/{\text{l}}_{s}}{\sum_{t\in S}{\text{n}}_{t}/{\text{l}}_{t}}$$

where, S denotes the whole strains set.

### Single-Nucleotide Polymorphism Calling

First, MetaPhlAn2 (Truong et al., 2015) with default setting was used to profile the microorganisms in each of these three samples. The identified microorganisms of each sample were collected as the microbial reference genomes of these three samples, respectively.^3^ Genome sequences of these microbial reference genomes were downloaded from the NCBI assembly database. Samples were mapped to their corresponding reference genomes with BWA (Li and Durbin, 2009). Only unique mapped reads were retained to increase the accuracy of SNP calling. Note that retaining only the unique mapped reads in the alignment process can avoid multiple gene copies issue. The sorted bam files were marked duplicates and filtered by Picard (Picard Toolkit, 2019). We combined Samtools (Li et al., 2009) and VarScan2 (Koboldt et al., 2012) together to reduce false positive results. Results from Samtools were filtered by VCFtools (Danecek et al., 2011) with parameters “+/d = 10/a = 4/Q = 15/q = 10/” and parameters for VarScan2 were “–min-coverage 10 –min-reads2 4 –min-var-freq 0.2 –*p*-value 0.05.” SNPs detected by both Samtools and VarScan2 were selected as tentative credible SNPs.

### Filtration Based on a Mixture Model of Depth Distribution

Noisy reads occurred in the sequencing process are primarily from substitution errors in Illumina platforms and lead to false positive SNPs. Considering general filtering thresholds of depth may not be applicable as sequencing depths increase, especially in ultra-deep sequencing data, we therefore attempted to set a variable threshold to filter false positive SNPs based on base depth distribution of strains (Supplementary Figure 1). Ideally, base depth distribution of a strain follows a normal distribution. Bases following other distributions except normal distribution may originate from sequencing errors or wrong matches due to homologous genes.

Hence, we constructed a mixture distribution model composed of an exponential distribution and a normal distribution to fit a sequenced base depth distribution of each strain. Genome depth is defined as the ratio of the total number of genome-mapped bases to the size of the corresponding genome (Sims et al., 2014). Python package Pomegranate was used to fit the corresponding mixture distribution model to data (Schreiber, 2017). Bases following the normal distribution were regarded to be derived from reads that mapped correctly to the strain genome, and bases following the exponential distribution were regarded to be derived from reads introduced by sequencing errors and homologous gene mismatches among different strains. To retain truly mapped reads as more as possible, we chose to keep at least 90% bases following the normal distribution. To exclude noisy reads, we chose to discard 50% bases following exponential distribution. To make a balance between keeping the majority of true assigned bases and filtering noise-derived bases, we determined to choose the smaller one of the two thresholds as the final filter threshold to retain at least 90% of bases following normal distribution. Furthermore, in the multiple sub-samples’ scenario, if the chosen threshold at one subsampling level is smaller than that at an adjacent lower subsampling level, we continued to use the threshold of the previous lower subsampling point, which actually only occurs in rare circumstances.

### Acquisition of Single-Nucleotide Polymorphism Function Profiles

Whether the SNPs are located in the coding region or non-coding region of the genome was determined according to the Gff file of the corresponding genome downloaded from NCBI. SNPs were annotated by SnpEff (Cingolani et al., 2012). Non-synonymous variants include “missense variant,” “start lost,” “stop gained” and “stop lost” types. Synonymous variants include “start retained variant,” “stop retained variant” and “synonymous variant.” The dN/dS ratio was calculated as an indicator of selection pressure acting on protein-coding DNA sequences (Mugal et al., 2014). Python package JoyPy^4^ was used to draw the mutation frequency distribution of dominant strains in each sample. The gene functions with enriched SNPs were also obtained by the gff file of the genome. Python package wordcloud (Oesper et al., 2011) was used to draw word cloud plots of gene functions with enriched SNPs. R package lmerTest was used to construct linear mixed effects models to test the relationship between relative abundance and SNPs. The models take sample as random effect and relative abundance as fixed effect (Kuznetsova et al., 2017).

### Strain-Level Population Genomics Analysis

To explore differences between SNPs identified from ultra-deep sequencing and that from conventional sequencing (6 GB), StrainPhlAn2 (Truong et al., 2017) was used to perform metagenomic strain-level population genomics analyses and phylogenetic analyses of major strains from metagenomic samples. In addition to the 6 GB subsamples and ultra-deep sequencing samples, we also used subsamples of 10 million, 50 million and 100 million reads as the input of StrainPhlAn2 to track the changes of the representative strain under different sequencing depths.

### Regression Models for Predicting Strain Single-Nucleotide Polymorphism Saturation

Cornell et al. proposed a term “damped increase” to account for the rate of diversification decreases to approach its upper limit (Cornell and Harrison, 2014). Hence, based on their perspectives, we used the ratio of SNP numbers (normalized to the interval [0,1]) to sequencing depth (normalized to the interval [0,1]) to determine whether a strain was saturated in ultra-deep samples. The ratio R is defined as follows:


$${\text{R}}_{i}=\frac{\left|Y_{i}-Y_{i-1}\right|}{X_{i}-X_{i-1}}$$

Where, *X_i_* represents the normalized depth of *i^th^* subsampling points, and *Y_i_* represents normalized SNP numbers of *i^th^* subsampling points. For a strain to be considered saturated, two conditions must be met: (1) as the sequencing depth deepens, Rs between two adjacent sub-sampling points gradually decreases; (2) R between the last two points is less than 0.1.

In order to predict the SNPs number of strains at their SNP saturation state, the sequencing coverage, sequencing depth, relative abundance, genome length, SNP number, SNP density and saturated SNP number of strains in the aforementioned subsamples and ultra-deep samples were used to construct a data set. Only saturated strains in our data were used here. The data set is divided into training set and test set according to the ratio of 4:1, and the python package scikit-learn (Pedregosa et al., 2011) was used to train a linear regression model and a random forest regression model, respectively. A grid search with 5-fold cross-validation strategy was utilized to achieve the best performances. The relative square error (RSE), relative absolute error (RAE) and coefficient of determination R-squared were used to evaluate the performance of models.

## Results

### A Framework to Obtain Reliable Metagenomic Single-Nucleotide Polymorphisms

**FIGURE 1:**
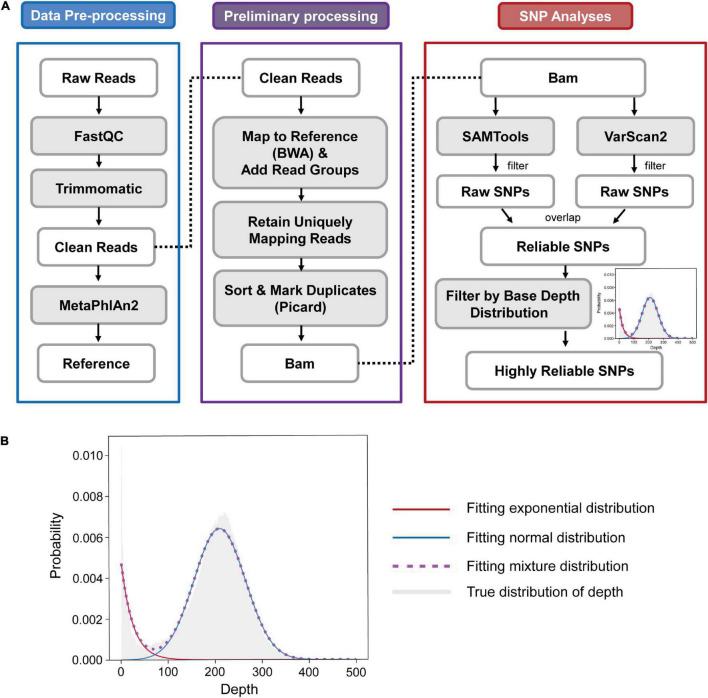
Workflow of reliable SNP calling. **(A)** A representation of the reliable SNP calling workflow, which contains three major parts: data pre-processing, preliminary processing and SNP analyses. The data pre-processing part is mainly to obtain clean data and appropriate reference genomes. The preliminary processing part contains several steps of sequence mapping to obtain unique alignments. The SNP analyses part contains SNP calling and filtering steps. **(B)** The mixture distribution model in the SNP analyses part is composed of an exponential distribution and a normal distribution fitting depth distribution of each strain to determine the filter threshold. The model was constructed to fit a sequenced base depth distribution of each strain, in which bases following the normal distribution were regarded to be derived from reads that mapped correctly to the strain genome, and bases following the exponential distribution were regarded to be derived from reads introduced by sequencing errors and homologous gene mismatches among different strains. The gray shaded part represents the true distribution of depth. The crimson line represents the fitting exponential distribution and the dodger blue line represents the fitting normal distribution. The medium orchid dashed line represents the fitting result of the mixture model.

To obtain highly reliable SNPs by ultra-deep sequencing, we established an accurate and effective framework to eliminate noisy and erroneous information when processing the data. The framework consists of three major parts, involving data pre-processing, preliminary processing and SNP analyses (Figure 1A). The data pre-processing part is mainly to control the quality of raw data to obtain clean data and appropriate reference genomes. The preliminary processing part contains several steps of sequence mapping to obtain unique alignments. The SNP analyses part contains SNP calling and filtering steps. Besides a two-way SNP calling procedure to acquire high-quality SNPs, we also utilized a bimodal distribution of base depths to remove potential false positive SNPs. The bimodal distribution consists of an exponential model for noisy reads and a normal model for truly assigned reads (Figure 1B and “Materials and Methods”). To check the performance of our framework, we benchmarked several SNP-calling strategies on simulated datasets. The results show that our framework exhibits the optimal result with the highest precision and the second highest sensitivity (Supplementary Material and Supplementary Figure 2). By using our framework, we can obtain highly reliable SNPs for the subsequent analysis.

### Ultra-Deep Sequencing Data and Metagenomic Single-Nucleotide Polymorphism Profiles of the Human Gut Microbiome

**FIGURE 2:**
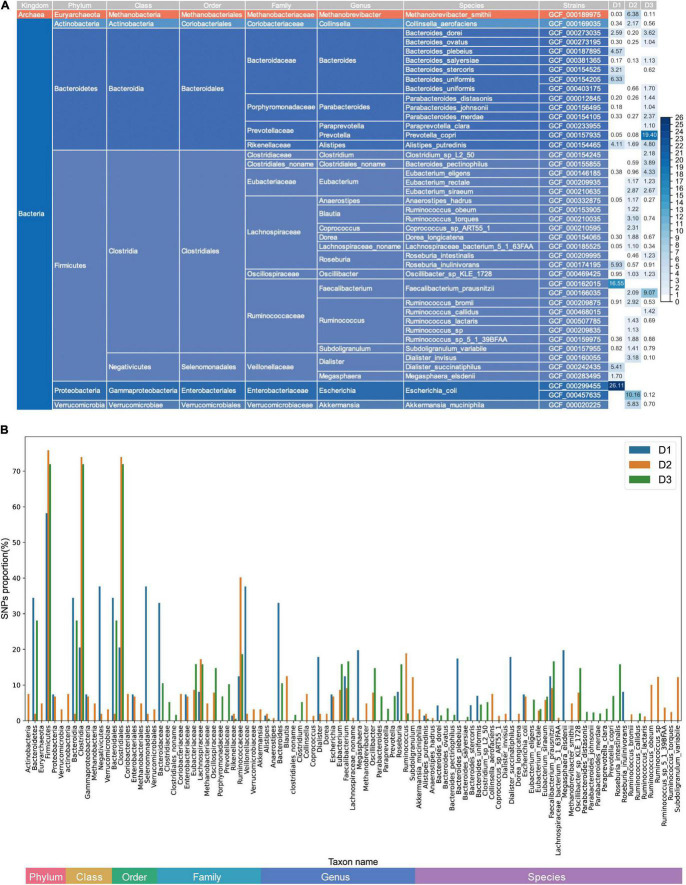
Relative abundances and SNP profiles of dominant strains in ultra-deep sequenced samples. **(A)** Relative abundances of major strains and their corresponding taxonomies. The right part is the relative abundance (%) heatmap of these dominant strains in the three samples. The deeper the color, the higher the relative abundance. The left part of the panel shows the taxonomy structure of the dominant strains. **(B)** Bar plot depicting the proportions of SNPs (in percentage) identified from microbes at different taxonomic levels. The proportions of SNPs equal the number of SNPs at a particular taxonomic level divided by the number of total SNPs. At one taxonomic level, the proportions in each sample sum to one.

In total, three ultradeep sequenced fecal samples were utilized in this study. They were labeled as D1, D2, and D3, with sequencing sizes of 437 GB (Gigabases), 786 GB and 754 GB, respectively. We identified the dominant strains (relative abundance >1%) of each sample (10 dominant strains in sample D1, 21 dominant strains in sample D2 and 19 dominant strains in sample D3). Details of the taxonomic levels of these strains are shown in Figure 2A. Among these 43 different dominant strains, 18 strains (41.86%) are shared by the three samples, which are prevalent strains in the human guts (Figure 2A). Community structures of the three samples are convergent at high taxonomic levels, but more diverse at low levels such as species level (Supplementary Figure 3).

Then, we identified reliable metagenomic SNP profiles of the dominant strains, namely, 693,338 SNPs in sample D1, 1,558,472 SNPs in sample D2, and 1,258,485 SNPs in sample D3. To investigate whether there exists any different SNP preference among difference microbes, we counted SNP numbers for each microbe as well as for higher taxonomic levels. The SNP proportions of microbial genomes within different taxonomic levels, namely the percentage of SNPs at different taxonomic levels, are shown in Figure 2B. Firmicutes have the most SNPs at the phylum level in all the three samples. Note that different taxonomic level contains different number of genomes, we therefore compared the average SNP number of genomes at certain taxonomic levels in the three samples. Firmicutes still have the most average SNPs in two of the three samples (D1, D3, Supplementary Figure 4). Firmicutes with relative abundance of about 30% contribute to 58.17, 75.85, and 71.93% SNPs of the dominant strains in the three samples, respectively. We conclude that as the common dominant bacteria in the human gut, Firmicutes’ long-term and continuous evolutionary pressure for adapting to the environment have led to a large number of SNPs.

As the taxonomic classification level varies from phylum to species, the differences of SNP proportions in the three samples further expand, which shows the heterogeneity among samples. In addition, we also studied the relationship between the number of SNPs and relative abundances of the dominant strains. As shown in Supplementary Figure 5, no significant correlation is indicated (Pearson correlation coefficient: 0.05, *p-*value = 0.74), suggesting that the number of SNPs and abundances of microbes might be independent. Linear mixed effects models using sample as random effect also support the non-correlation (*p-*value = 0.74).

### Metagenomic Single-Nucleotide Polymorphism Function Profiles by Ultra-Deep Sequencing in the Human Gut

**FIGURE 3:**
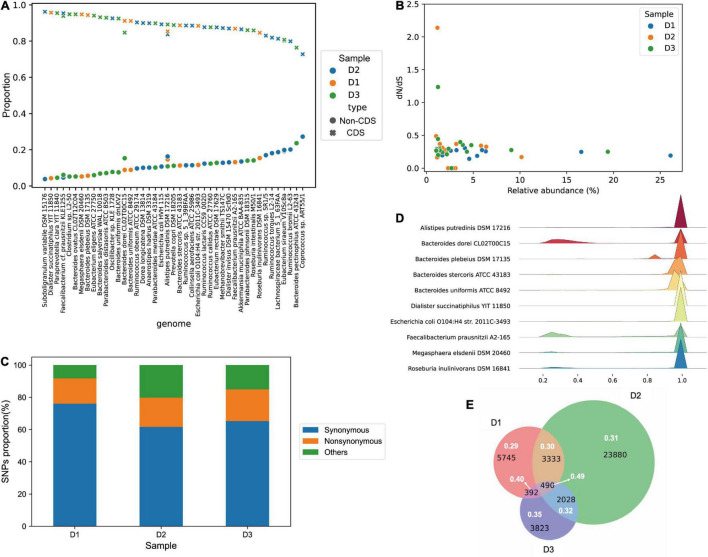
SNP function profiles of dominant strains in ultra-deep sequencing samples. **(A)** Proportions of the CDS-SNPs and the non-CDS-SNPs in each sample. For each dominant strain in samples, the proportion of the CDS-SNPs represents the proportion of SNPs located in the coding region, and the proportion of the non-CDS-SNPs represents the proportion of SNPs located in the non-coding region. **(B)** Relationships between the dN/dS ratio and relative abundances of dominant strains in each sample. The dN/dS ratio equals the number of non-synonymous SNPs divided by the number of synonymous SNPs. **(C)** Proportions of different types of the cumulative SNPs from all dominant strains in the ultra-deep sequencing samples. **(D)** Allele frequencies of SNPs of dominant strains in sample D1. The *X*-axis represents the allele frequencies of SNPs, and *Y*-axis shows the name of dominant strains. **(E)** Venn diagrams of SNPs of strain *Alistipes putredinis* DSM 17216 in the three samples. Black numbers represent the number of SNPs, and white numbers represent the dN/dS ratio of these SNPs.

To evaluate the functional impacts of metagenomic SNPs, we checked whether the SNPs are located in the coding region (CDS-SNPs) or the non-coding region (non-CDS-SNPs) of genomes. As shown in Figure 3A, for each strain, most of the SNPs are located in the coding region. After sorting the strains by proportions of SNPs in non-coding regions, the top nine strains (*Roseburia intestinalis* M50/1, *Roseburia inulinivorans* DSM 16841, *Ruminococcus* sp. SR1/5, *Ruminococcus torques* L2-14, *Lachnospiraceae bacterium* 5_1_63FAA, *Eubacterium siraeum* V10Sc8a, *Ruminococcus bromii* L2-63, *Bacteroides pectinophilus* ATCC 43243 and *Coprococcus* sp. ART55/1) with relatively high proportions of SNPs in non-coding regions, all originate from the order *Clostridiales*. *Clostridiales* are considered to be the most active microbial components in the intestinal environment of healthy adults, including polysaccharide-decomposing bacteria, which contribute greatly to the production of short-chain fatty acids in the intestine (Chinda et al., 2004).

In order to further study the functional changes, SNPs were annotated and classified into non-synonymous variants, synonymous variants and other variants. The dN/dS ratio of each strain was also calculated, which is an indicator of the selective pressure acting on protein-coding DNA sequences (Mugal et al., 2014). In addition, the relationship between the dN/dS ratio and strain relative abundance was also analyzed, as shown in Figure 3B. The dN/dS ratio of most of the strains varies between 0 and 0.5; the correlation between the dN/dS ratio and relative abundance is low (Pearson correlation coefficient = −0.05, *p*-value = 0.75). Further, the cumulative SNPs from all dominant strains in the three samples were calculated. As shown in Figure 3C, synonymous variants accounted for the vast majority (D1: 76.06%, D2: 61.70%, D3: 65.25%), and non-synonymous variants accounted for a small proportion (D1: 15.73%, D2: 18.09%, D3: 19.73%); the dN/dS ratio of sample D1, D2, and D3 are 0.21, 0.29, and 0.30, respectively.

Subsequently, we further investigate genes with enriched SNPs, namely genes have more SNPs compared to background. A word cloud of these gene functions is shown in Supplementary Figure 6. For sample D1, the top five SNP-enriched functions are TonB-dependent receptor, ATP-binding cassette (ABC) transporter ATP-binding protein, ABC transporter permease, and major facilitator superfamily (MFS) transporter. Among them, the ABC transporter uses the energy of ATP binding and hydrolysis to transport various substrates across the cell membrane. In prokaryotes, it mediates the absorption of nutrients into cells. Substrates that can be transported include ions, amino acids, peptides, sugars, and most other hydrophilic molecules. The bacterial ABC transporter is essential for cell viability, virulence and pathogenicity. ABC transporter permease is also involved in transmembrane transport (Schneider and Hunke, 1998; Davidson et al., 2008). In addition to these proteins related to ABC transport, the SNP-enriched functions in sample D1 are mainly focused on basic processes of life activities related to transmembrane transport, ATP binding and DNA binding. Interestingly, we found that the functions of SNP-enriched genes in sample D2 and D3 have high similarities with D1, both involving ABC transporter ATP-binding protein, ABC transporter permease, MFS transporter, AAA family ATPase, etc. SNPs are enriched in genes associated with basic life activities, which might indicate that the genes are related to the adaptation to environment and have strong interaction with the environment.

Furthermore, we studied the allele frequencies of SNPs by ultra-deep sequencing (Figure 3D and Supplementary Figure 7). The allele frequencies of most SNPs from different strains are near 1.0, indicating that most of the mutation sites are homozygous. A few sites are heterozygous, which might be a result of genetic drift in the community.

Since the three samples are from two different continents, a deep comparison needs to be performed to investigate the SNP differences among samples under the ultra-deep sequencing scenario. Because only one dominant strain was found present in all the three samples, namely *Alipipes putredinis* DSM 17216, we conducted an in-depth study on this strain and compared SNPs of it in different samples. Among the SNPs of *A. putredinis* DSM 17216, synonymous variants accounted for the vast majority (D1: 65.38%, D2: 63.86%, D3: 66.02%), and non-synonymous variants accounted for a small portion (D1: 19.98%, D2: 19.86%, D3: 23.15%); the dN/dS ratios of *A. putredinis* DSM 17216 in sample D1, D2, and D3 are 0.31, 0.31, and 0.35, respectively (Figure 3E). As shown in Figure 3E, the three samples have only 490 SNPs in common for the shared strain, and most of the remaining SNPs are unique to each sample, which may imply that the same strain has different mutation responses towards different environmental pressures.

We divided the SNP sites of the strain into different categories according to whether they are shared among samples (at least two samples) or not. Interestingly, the dN/dS ratio of the shared SNPs is relatively high, especially the dN/dS ratio of the 490 three-samples-shared SNPs (0.49) greatly exceeds that of two-samples-shared (0.30, 0.40 and 0.32) SNPs and each sample’s exclusive SNPs (0.29, 0.35 and 0.31, respectively) (Chi-square test, *p* = 1.24e-5). The 490 SNPs are mainly located in genes with functions of catalytic enzymes, transferases, recombination integrases, and others. Note that these genes are related with DNA recombination (Kwon et al., 1997), DNA integration, DNA binding processes (Esposito and Scocca, 1997), ATP binding, potassium ions binding and sodium ions binding (Miyauchi et al., 2013), which indicates the response of these important genes to environmental pressures.

### Comparison of Single-Nucleotide Polymorphisms Identified From Ultra-Deep Sequencing and Conventional Sequencing Data

**FIGURE 4:**
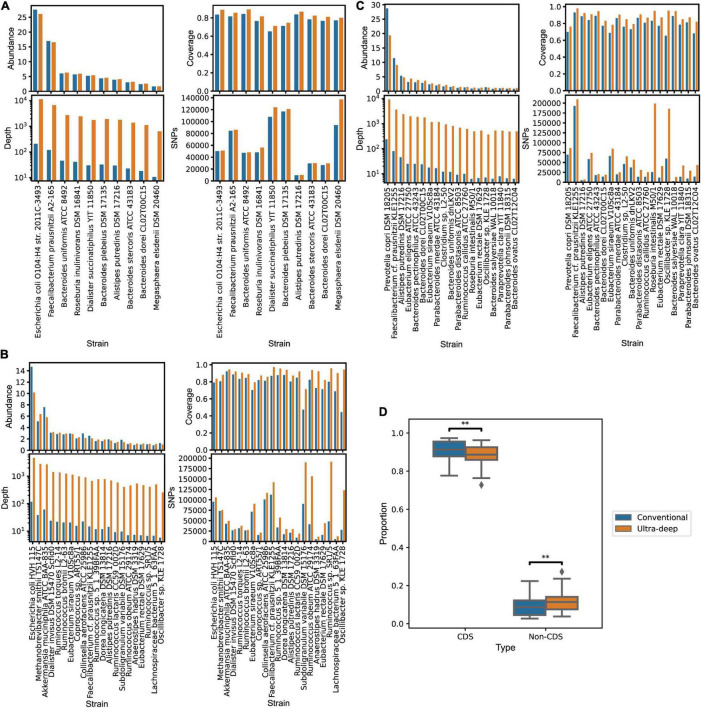
Comparison of SNPs identified from ultra-deep sequencing and conventional sequencing data (6G). Relative abundances, sequencing coverages, depths, and SNP numbers of dominant strains are compared in sample D1 **(A)**, D2 **(B)** and D3 **(C)**. Orange bars represent the ultra-deep sequencing data and blue bars represent the conventional sequencing data. **(D)** The proportion of the cumulative CDS-SNPs and the non-CDS-SNPs of dominant strains from ultra-deep sequencing (Ultra-deep) and conventional sequencing data (Conventional).

In order to investigate whether additional sequencing depth will contribute to new discoveries of SNPs analysis, we compared the SNPs from ultra-deep sequencing data with those from conventional sequencing data. The conventional sequencing size of 6 GB of one sample was recommended when studying the human intestinal metagenomics (Liu et al., 2021). We randomly down-sampled the three ultra-deep metagenomic sequencing samples into 6 GB sequencing subsamples, and studied the dominant strains’ relative abundances, sequencing coverages, depths, and SNPs amounts, as shown in Figures 4A–C. We found that the relative abundance of dominant strains did not change much on the whole, which shows that the conventional sequencing size is suitable for the study of species composition of the gut microbe community. The sequencing coverage of these dominant strains by ultra-deep sequencing has increased to varying degrees and the sequencing depth of strains by ultra-deep sequencing has significantly improved compared with that by conventional sequencing size. As the sequencing depths increases, the numbers of SNPs also increase to varying degrees, and increases in certain strains are particularly huge.

For strains with particularly high increases in SNPs in ultra-deep sequencing data, we suspected that additional strains are detected due to the increased sequencing content. Hence, we used StrainPhlAn2 to track the changes of the representative strain under different sequencing depths (see “Materials and Methods”). For sample D1, we identified much more SNPs of *Megasphaera elsdenii* (relative abundance: 1.63% from 6G data, 1.70% from ultra-deep sequencing data) from ultra-deep sequencing data than from conventional sequencing data. From the phylogenetic tree in Supplementary Figure 8A, the representative strain from 6 GB data is closer to the reference genome, whereas the strain from ultra-deep sequenced D1 is farther from the reference genome. Results of sample D2 and D3 are also shown in Supplementary Figures 8B,C with *Ruminococcus obeum* (relative abundance: 1.08% from 6G data, 1.22% from ultra-deep sequencing data) and *Roseburia intestinalis* (relative abundance: 0.93% from 6G data, 1.23% from ultra-deep sequencing data) as examples, respectively. These results suggest that deep sequencing of metagenomic samples could lead to detection of more strains, which could be overlooked by conventional shallow sequencing.

We also found that the SNPs from ultra-deep sequencing data has a lower proportion of CDS-SNPs and a higher proportion of non-CDS SNPs, compared to those from conventional sequencing data (Figure 4D, *p* = 3.2e-3, Mann–Whitney U rank sum test). To further investigate the increased SNPs due to the deeper sequencing, we checked the functions of the genes where newly detected SNPs locate. More functional genes with SNPs detected in the deep sample were involved, as shown in Supplementary Figures 8D–F. The newly involved genes are mainly concentrated in plasmid replication initiator protein, phage integration family enzymes, kinases, transferases and phosphatases, etc. These proteins participate in essential activities such as ATP binding, mismatch repair, methylation process, catalysis of inorganic salt water hydrolysis and lipid metabolism. The results indicate deeper sequenced data could detect more functionally important SNPs. This also implies that the conventional sequencing size is not enough to support a comprehensive study of SNPs.

### Single-Nucleotide Polymorphism Profile Variations Caused by Sequencing Depth Difference

**FIGURE 5:**
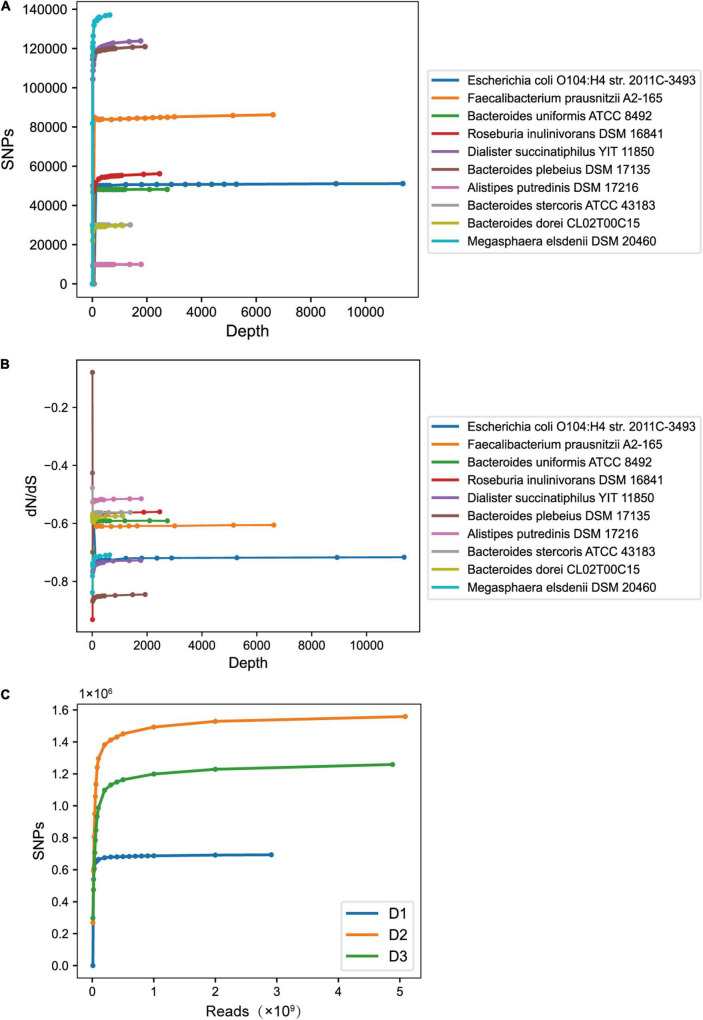
The trend of SNP number **(A)** and dN/dS ratio **(B)** changes of dominant strains from sample D1 caused by the sequencing depth increasement. The dN/dS ratio is plotted on the log scale to show the changing trend of dN/dS more clearly. **(C)** The trend of cumulative SNP numbers from all dominant strains in sample level as sequencing size increases.

Besides the conventional sequencing size of 6 GB, we also studied the SNP profiles in data with varying depths (see “Materials and Methods”). We first focused on SNP profile changes of single strains as the sequencing depth increases. As shown in Figure 5A, as the sequencing depth of the strain increases, the number of SNPs rises up quickly at first and then tends to be stable (Supplementary Figure 9A). We also studied the changes of the strain’s dN/dS ratio as the sequencing depth increased. Similarly, the dN/dS ratios fluctuated greatly at the beginning, and gradually stabilized with the sequencing depth increasing (Figure 5B and Supplementary Figure 9B). This indicates that in metagenomic SNPs studies, the dN/dS ratio inferred from the data cannot reflect the true selection pressure when the sequence depth is not enough. We also checked the trend of cumulative SNPs from all dominant strains as the sequenced read counts increase in sample level (Figure 5C).

**FIGURE 6:**
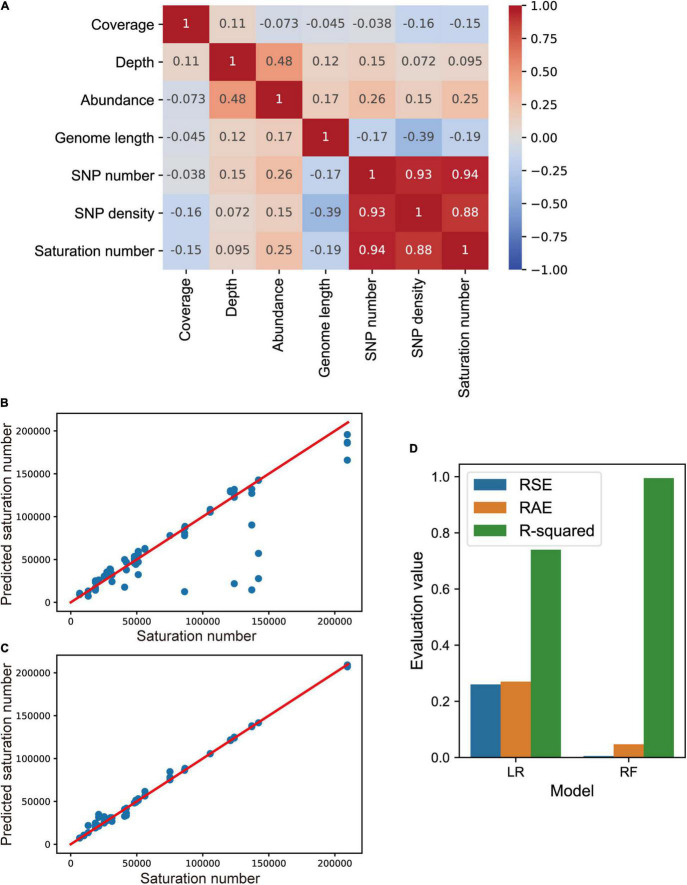
SNP saturation prediction of strains using machine learning models. **(A)** Correlation heatmap among sequencing coverage, sequencing depth, relative abundance, genome length, SNP number, and SNP density of dominant strains. The SNP density equals the SNP numbers of a strain divided by the covered length of the genome. The predicted outcomes of linear regression (LR) model **(B)**, and random forest (RF) regression model **(C)** are presented. The *X*-axis represents the SNP saturation number of strains, and the *Y*-axis represents the predicted SNP saturation number. **(D)** The relative square error (RSE), relative absolute error (RAE) and coefficient of determination R-squared values from LR and RF models are compared.

Furthermore, we investigated whether we can use machine learning models to predict the overall SNP numbers of strains given sequencing depths and other parameters from an under-sequenced sample (see “Materials and Methods”). We used current sequencing coverage, current sequencing depth, current relative abundance, genome length, current SNP number, and current SNP density of strains in subsamples to predict the final saturated SNP number for each strain. Data from subsamples were collected. First, the correlations between different variables were compared. The SNP saturation number has high correlations with current SNP number and current SNP density, while having low correlations with current sequencing coverage, current sequencing depth, current relative abundance, and genome length (Figure 6A). Then we used linear regression (LR) model and random forest (RF) regression model to predict the saturated SNP number, and the prediction results are shown in Figures 6B,C.

Relative square error (RSE), relative absolute error (RAE), and coefficient of determination R-squared were used to evaluate the performance of models. The smaller the RSE and the RAE, the better the model predicts; the higher the R-squared, the better the model fits the data. As shown in Figure 6D, the random forest regression model fits better, with R-squared coefficient reaching 0.99. Variable feature importances in the RF regression model is 0.12, 0.01, 0.09, 0.04, 0.31 and 0.43, respectively, which are in consistence with the correlations among parameters. We can use this model named SNPsnp (SNP saturation number prediction) to predict the saturated SNP number of a strain, given its sequencing coverage, sequencing depth, relative abundance, SNP number and SNP density in a shallow-sequenced sample. A test sample could be sequenced in an affordable depth first to determine if more data is needed. The model SNPsnp is available on GitHub.^5^

## Discussion

This research is a deep study of metagenomic samples by ultra-deep sequencing in the human gut. We investigate the gut microbial communities of the shallow-sequenced and ultra-deep sequenced samples, provides a framework for reliable SNP identification by ultra-deep sequencing, conducts SNP analysis and obtains SNP profiles by ultra-deep sequencing. SNP profiles from ultra-deep sequencing and conventional sequencing data were compared. Also, SNP changes with sequencing depth increasing were investigated and a machine learning model was constructed to guide researchers determine the optimal and affordable sequencing depth for their projects.

According to our results, the conventional sequencing sizes of 6 GB for metagenomics studies for the human gut microbiome is not enough to support a comprehensive study of metagenomic SNPs and related downstream analysis. We found that compared with that from conventional sequencing size, SNPs identified from ultra-deep sequencing involved more important genes and functions. The results by StrainPhlAn2 suggest that deep sequencing of metagenomic samples could lead to detection of rare strains, which might be overlooked by conventional shallow sequencing. Further work is needed to solve the limitation of our work. The limitations include the sensitivity reduction of SNP calling caused by unique mapping, and the cost-intensive preliminary sequencing requirement for sequencing depth’s determination.

Nowadays, metagenomic research has moved from species level to the high-resolution strain level, in which SNP identification plays an important role. By using our predictive model, the appropriate sequencing depths could be estimated using test samples prior to metagenomic variations investigation. Sufficient depth of sequencing can promote accurate analysis of metagenomic SNPs and further facilitate discovering accurate associations between metagenomic SNPs and diseases. Though more experiments need to be conducted to validate our work, our findings could provide valuable perspectives for future studies on metagenomic variations of the human gut or other environments, and further promote strain-level researches.

## Data Availability Statement

The raw sequence data reported in this manuscript have been deposited in the Genome Sequence Archive (Chen T. et al., 2021) in National Genomics Data Center (CNCB-NGDC Members and Partners, 2022), China National Center for Bioinformation/Beijing Institute of Genomics, Chinese Academy of Sciences (GSA: CRA005603) that are publicly accessible at https://ngdc.cncb.ac.cn/gsa.

## Ethics Statement

The studies involving human participants were reviewed and approved by Ethics Committee of Yongkang First People’s Hospital. The patients/participants provided their written informed consent to participate in this study. Written informed consent was obtained from the individual(s) for the publication of any potentially identifiable images or data included in this article.

## Author Contributions

XY, YC, and PL: conceptualization. YC, PL, and ZH: methodology. PL, YC, and SA: software. YC, PL, and GD: validation. PL, YC, and CF: formal analysis. PL and YC: investigation. RL: data curation. FX: provision of samples. PL: writing—original draft preparation. XY and YC: writing—review and editing and supervision. PL and SH: visualization. YC and CF: project administration. XY and FX: funding acquisition. All authors have read and agreed to the published version of the manuscript.

## Conflict of Interest

The authors declare that the research was conducted in the absence of any commercial or financial relationships that could be construed as a potential conflict of interest.

## Publisher’s Note

All claims expressed in this article are solely those of the authors and do not necessarily represent those of their affiliated organizations, or those of the publisher, the editors and the reviewers. Any product that may be evaluated in this article, or claim that may be made by its manufacturer, is not guaranteed or endorsed by the publisher.

## Footnotes

1
http://www.bioinformatics.babraham.ac.uk/projects/fastqc/

2
https://github.com/labomics/SNPsnp/tree/main/SNP_calling/step1_qc

3
https://github.com/labomics/SNPsnp/tree/main/SNP_calling

4
https://github.com/leotac/joypy

5
https://github.com/labomics/SNPsnp
