# Supplementary material for: Towards Strain-Level Complexity: Sequencing Depth Required for Comprehensive Single-Nucleotide Polymorphism Analysis of the Human Gut Microbiome
Source: Front Microbiol. 2022 May 5;13:828254. doi: 10.3389/fmicb.2022.828254 (PMC9119422; doi:10.3389/fmicb.2022.828254)
Supplement: Supplementary file 1 [file Data_Sheet_1.zip › supplementary/Supplementary_material.pdf]

Supplementary Material

1 Supplementary methods

Simulation datasets generation

To generate simulation data, we considered two different scenarios: a simple strain composition scenario and a complex strain composition scenario (Andreu-Sánchez et al., 2021; Zhu et al., 2021). The first scenario contains 5 strains (*Faecalibacterium prausnitzii*: GCF_000166035, *Roseburia hominis*: GCF_000225345, *Bacteroides fragilis*: GCF_000009925, *Prevotella copri*: GCF_000157935 and *Fusobacterium nucleatum*: GCF_000158255) from predominant bacteria in the ZymoBIOMICS Gut Microbiome Standard. To generate synthetic data, we randomly generated 11,505 SNPs for the first reference genomes. The second scenario contains 142 strains, which refers to the actual situation, namely, using the reference genomes of our sample D1. And we randomly generated 11,120 SNPs for the second reference genomes in one of these strains. InSilicoSeq (Gourlé et al., 2019) was used to generate a simulated dataset of 20 million Hiseq paired-end reads (the conventional sequencing size) based on the mutated reference genomes, with parameters "iss generate --model HiSeq --abundance lognormal". Error model 'HiSeq' was chosen to generate reads from the Illumina HiSeq instrument.

Comparison on metaSNV, inStrain, VarScan2, SAMTools and our SNP calling pipeline

Based on the cleaned sorted bam files and the reference (not-mutated) genomes, SNP calling was performed by metaSNV, inStrain, VarScan2, SAMTools and our pipeline. Because we focus on the credible SNP acquisition, we only compare the SNP calling results rather than the downstream outputs of these tools.

metaSNV is a SNP calling pipeline scaling well with metagenomic datasets and is capable of handling large multi-species references. metaSNV identifies SNPs using SAMTools mpileup and mappings of short reads against processed bam files (Costea et al., 2017). inStrain is a tool for sensitive SNP detection that allows highly accurate genome comparisons (Olm et al., 2021). SAMTools is a commonly used pipeline (high accuracy and easy use) to call SNPs (Li et al., 2009). VarScan2 (Koboldt et al., 2012) is very robust in adjusting thresholds such as coverage and minimum allele frequency, which makes it advantageous for the detection of low-allele-frequency variants in high-depth datasets. The default settings were applied in metaSNV, inStrain, VarScan2 and SAMTools.

Sensitivity and precision calculation

To quantitatively evaluate the performance of SNP calling tools, we assessed their performance from two aspects which were calculated as follows.

$$\text{Sensitivity} = \frac{TP}{TP+FN}$$

In which, TP: true positive; FN: false negative.

$$\text{Precision} = \frac{TP}{TP+FP}$$

In which, TP: true positive; FP: false positive.

True positive (TP) represents SNP sites that are correctly identified by the calling procedure. False positive (FP) represents non-SNP sites that are wrongly identified as SNPs. True negative (TN) represents sites that are correctly identified as non-SNP sites. False negative (FN) represents SNPs that are wrongly identified as non-SNP sites.

References

Andreu-Sánchez, S., Chen, L., Wang, D., Augustijn, H. E., Zhernakova, A., and Fu, J. (2021). A Benchmark of Genetic Variant Calling Pipelines Using Metagenomic Short-Read Sequencing. *Frontiers in Genetics* 12. doi:10.3389/fgene.2021.648229.

Costea, P. I., Munch, R., Coelho, L. P., Paoli, L., Sunagawa, S., and Bork, P. (2017). metaSNV: A tool for metagenomic strain level analysis. *PLoS One* 12, e0182392. doi:10.1371/journal.pone.0182392.

Gourlé, H., Karlsson-Lindsjö, O., Hayer, J., and Bongcam-Rudloff, E. (2019). Simulating Illumina metagenomic data with InSilicoSeq. *Bioinformatics* 35, 521–522. doi:10.1093/bioinformatics/bty630.

Koboldt, D. C., Zhang, Q., Larson, D. E., Shen, D., McLellan, M. D., Lin, L., et al. (2012). VarScan 2: Somatic mutation and copy number alteration discovery in cancer by exome sequencing. *Genome Research* 22, 568–576. doi:10.1101/gr.129684.111.

Li, H., Handsaker, B., Wysoker, A., Fennell, T., Ruan, J., Homer, N., et al. (2009). The Sequence Alignment/Map format and SAMtools. *Bioinformatics* 25, 2078–2079. doi:10.1093/bioinformatics/btp352.

Olm, M. R., Crits-Christoph, A., Bouma-Gregson, K., Firek, B. A., Morowitz, M. J., and Banfield, J. F. (2021). inStrain profiles population microdiversity from metagenomic data and sensitively detects shared microbial strains. *Nat Biotechnol* 39, 727–736. doi:10.1038/s41587-020-00797-0.

Zhu, X., Qin, J., Tan, C., and Ning, K. (2021). The seasonal changes of the gut microbiome of the population living in traditional lifestyles are represented by characteristic species-level and functional-level SNP enrichment patterns. *BMC Genomics* 22, 83. doi:10.1186/s12864-021-07372-0.

2 Supplementary Figures and Tables

2.1 Supplementary Figures

Figure S1. Distributions of the base depths of two representative strains (A and B). The blue shaded part represents the distribution of base depths. Base depth indicates the covered reads count for each genome site.

Figure S2. The performances (sensitivity and precision) of different tools (metaSNV, inStrain, VarScan2, SAMTools and our pipeline) based on the truth datasets from simple scenario (A) and complex scenario (B).

Figure S3. Relative abundance of major strains at the phylum (A), class (B), order (C), family (D), genus (E) and species (F) level in the ultra-deep sequencing samples.

Figure S4. Bar plot depicting the average SNPs identified from microbes at different taxonomic levels. The average SNPs equal the number of SNPs at a particular taxonomic level divided by the genome number at that level.

Figure S5. Relationships between SNP numbers and relative abundances of dominant strains in each ultra-deep sequencing sample.

Figure S6. Word cloud plots of gene functions with enriched SNPs of dominant strains in each ultra-deep sequencing sample (A, B, C for D1, D2 and D3, respectively). The size of words indicates the SNPs enrichment degree of genes.

Figure S7. Allele frequencies of SNPs of dominant strains in sample D2 (A) and D3 (B) respectively. The X-axis represents the allele frequency of SNPs, and Y-axis shows the name of dominant strains.

Figure S8. Phylogenetic tree of the identified *Megasphaera elsdenii* genomes in sample D1 (A), *Ruminococcus obeum* genomes in sample D2 (B) and *Roseburia intestinalis* genomes in sample D3 (C). The "6G" represents subsamples of the conventional sequencing size 6G, "100m", "500m" and "1b" represent subsamples of 10 million, 50 million and 100 million reads, respectively. The Venn diagrams show numbers of SNP-enriched genes from conventional sequencing size (6G) data and ultra-deep sequencing data of sample D1 (D), D2 (E) and D3 (F), respectively.

Figure S9. The trend of SNP number (A) and dN/dS (B) changes of dominant strains from sample D2 and D3 caused by the sequencing depth increasement. The dN/dS ratio is plotted on the log scale to show the changing trend of dn/ds more clearly.
