# Supplementary figures and images for: Towards Strain-Level Complexity: Sequencing Depth Required for Comprehensive Single-Nucleotide Polymorphism Analysis of the Human Gut Microbiome
Source: Front Microbiol. 2022 May 5;13:828254. doi: 10.3389/fmicb.2022.828254 (PMC9119422; doi:10.3389/fmicb.2022.828254)

A

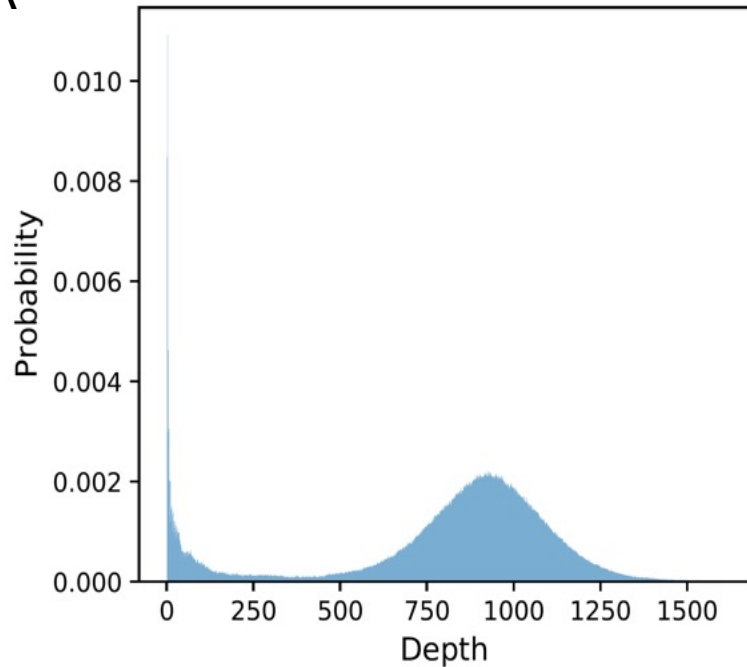

B

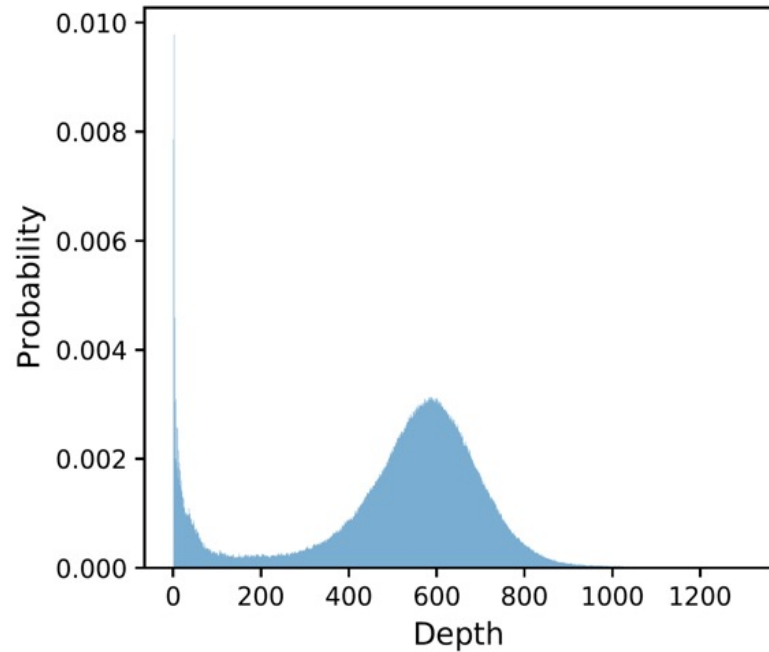

Supplement: Supplementary file 1 [file Data_Sheet_1.zip › supplementary/FigureS1.pdf]

A

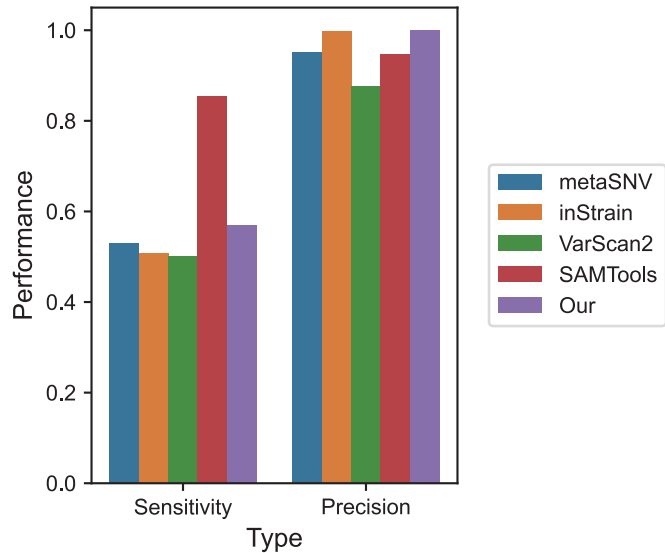

B

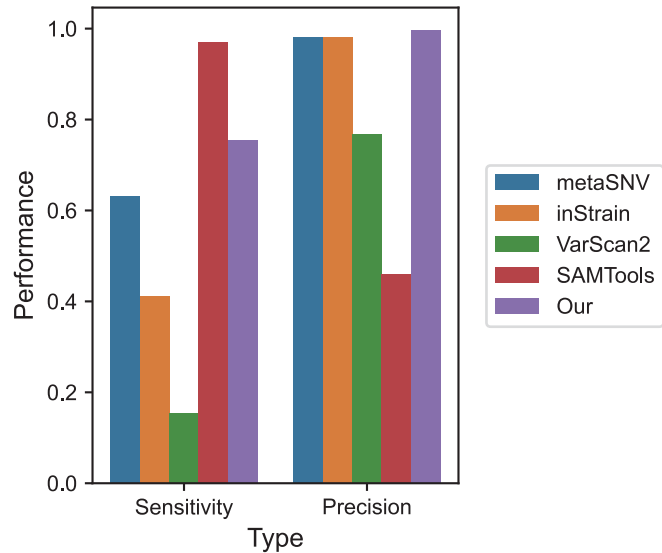

Supplement: Supplementary file 1 [file Data_Sheet_1.zip › supplementary/FigureS2.pdf]

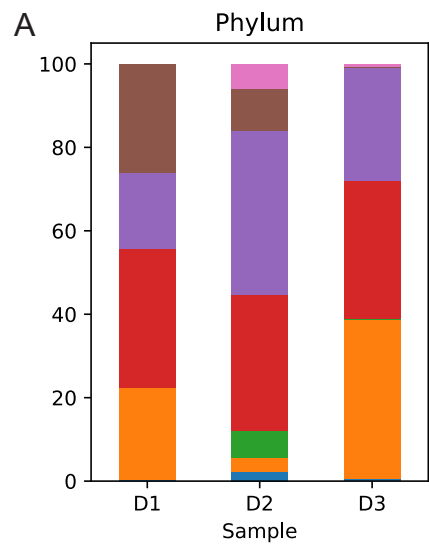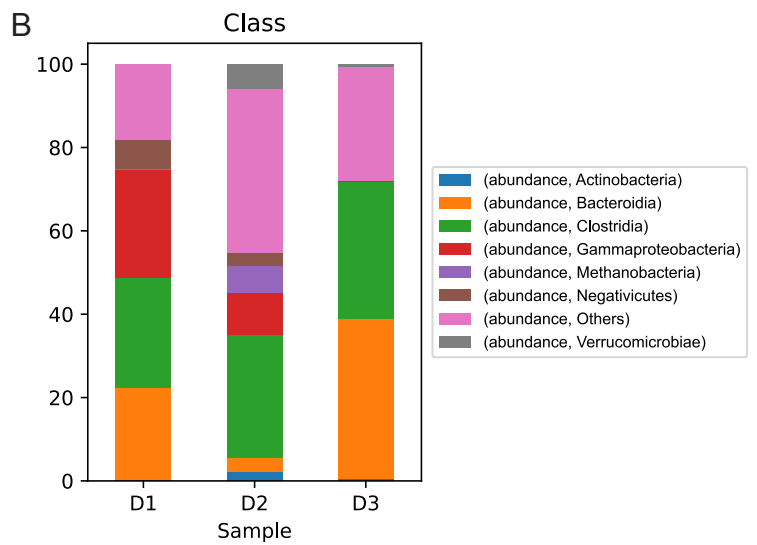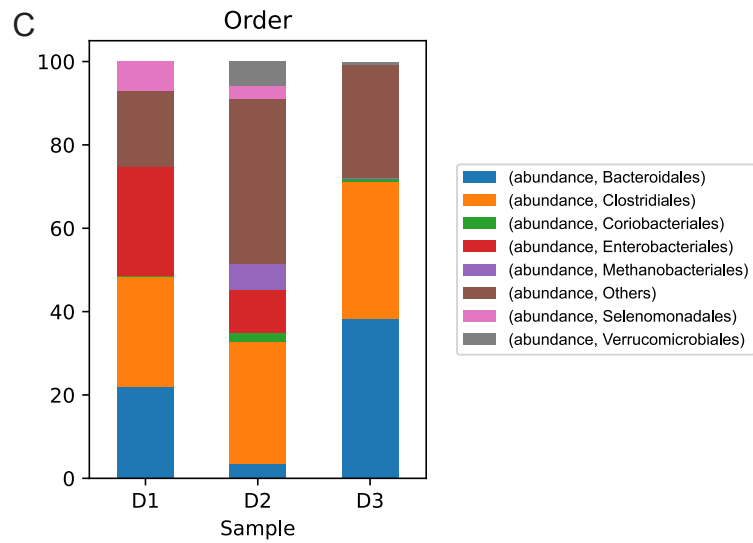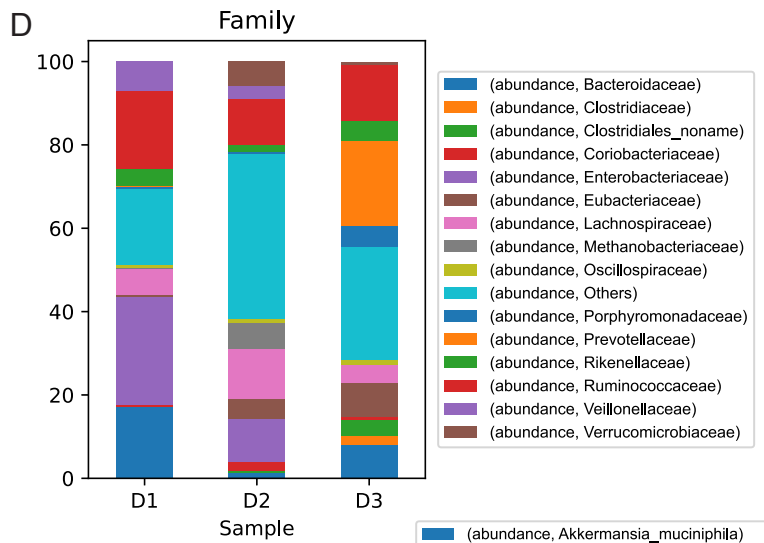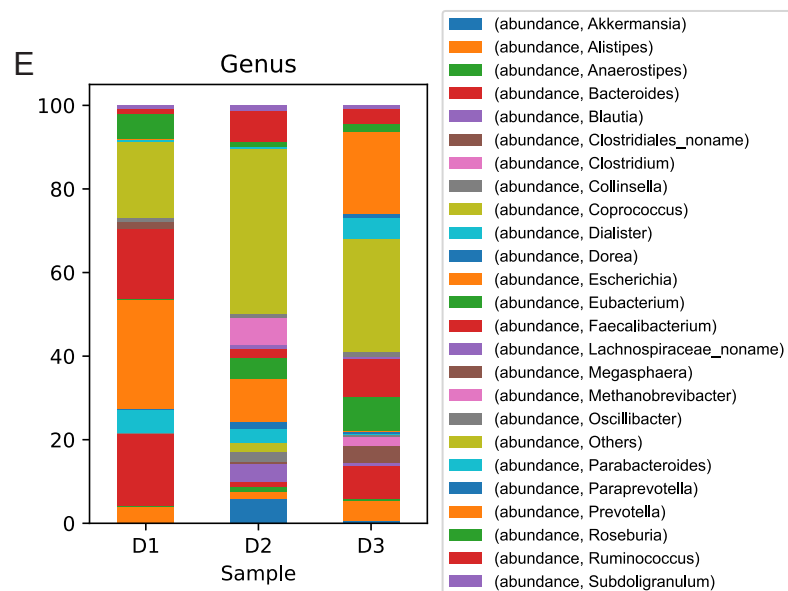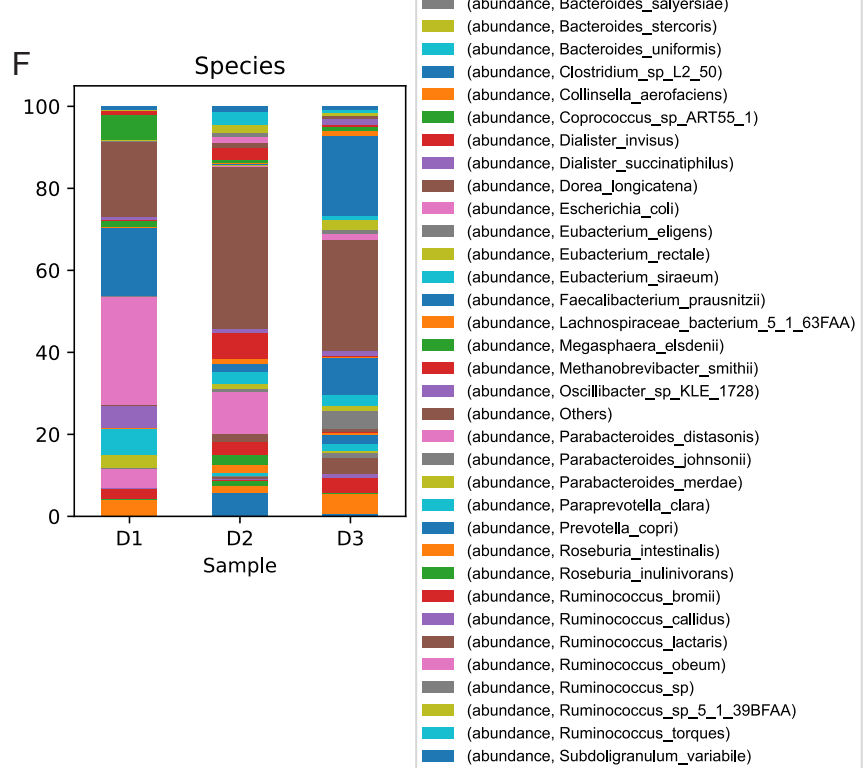

Supplement: Supplementary file 1 [file Data_Sheet_1.zip › supplementary/FigureS3.pdf]

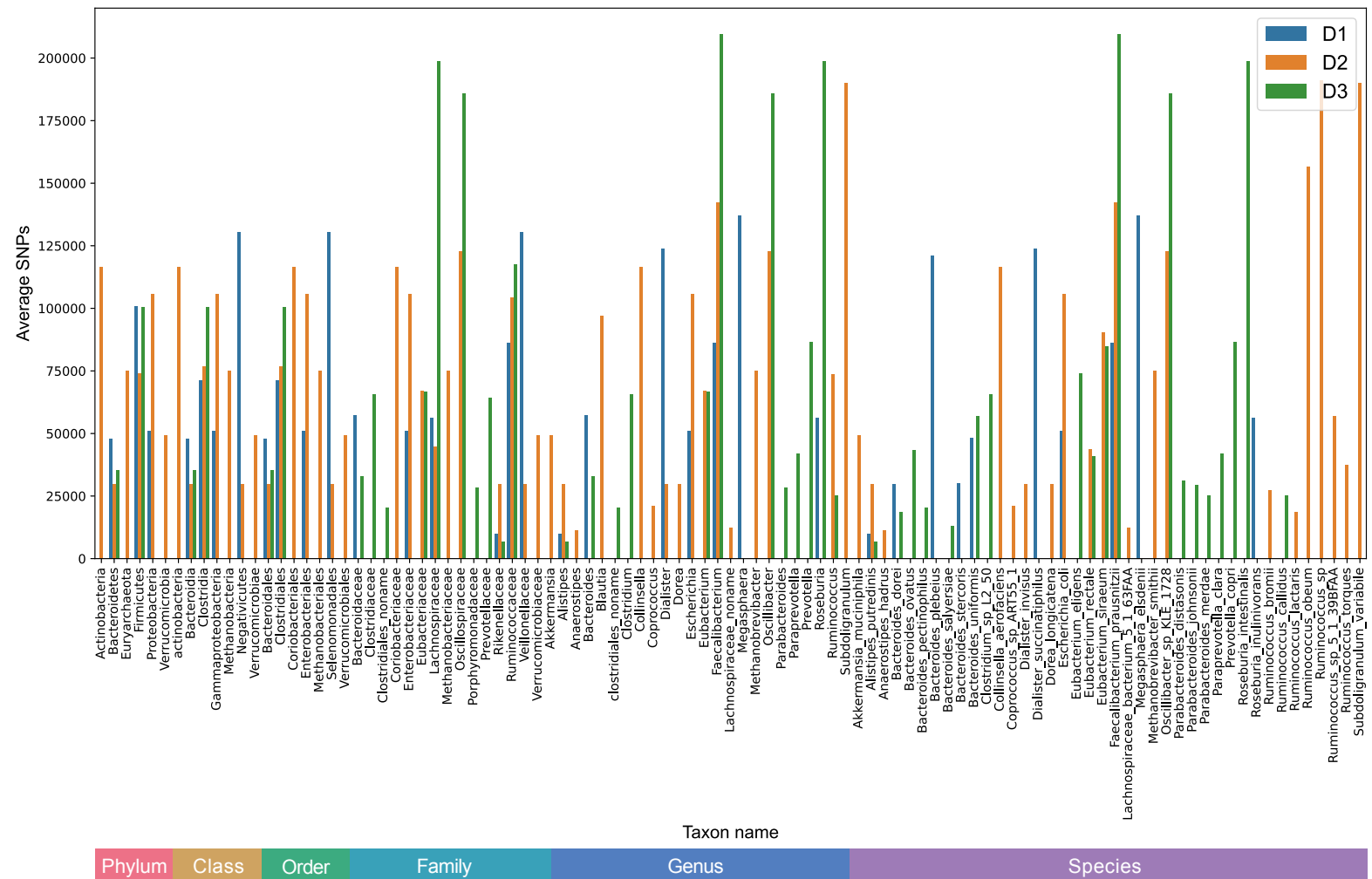

Supplement: Supplementary file 1 [file Data_Sheet_1.zip › supplementary/FigureS4.pdf]

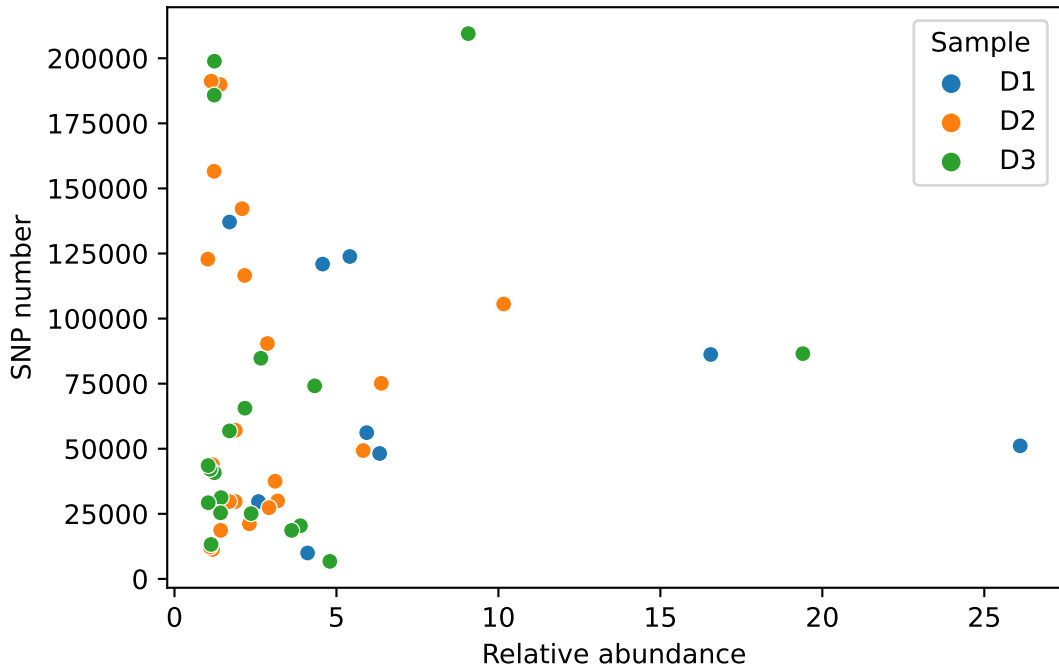

Supplement: Supplementary file 1 [file Data_Sheet_1.zip › supplementary/FigureS5.pdf]

A

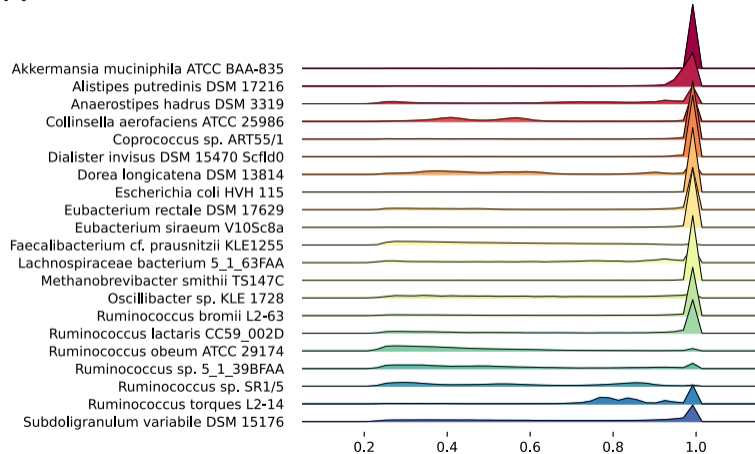

B

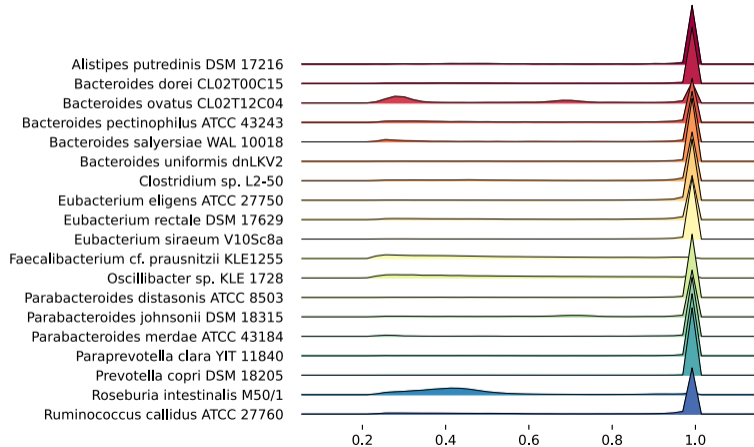

Supplement: Supplementary file 1 [file Data_Sheet_1.zip › supplementary/FigureS7.pdf]

A

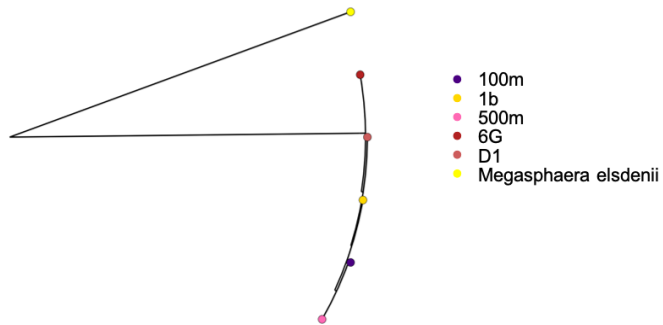

B

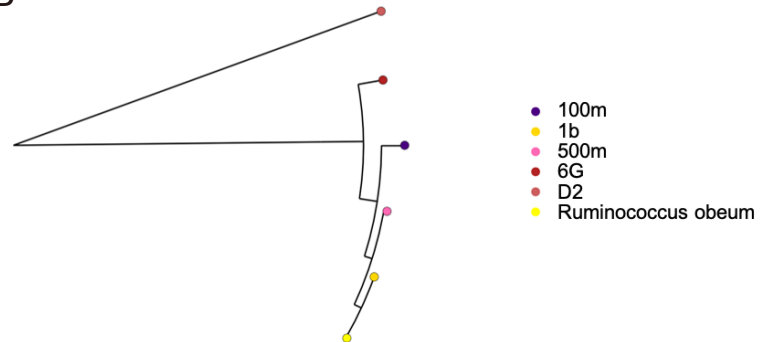

C

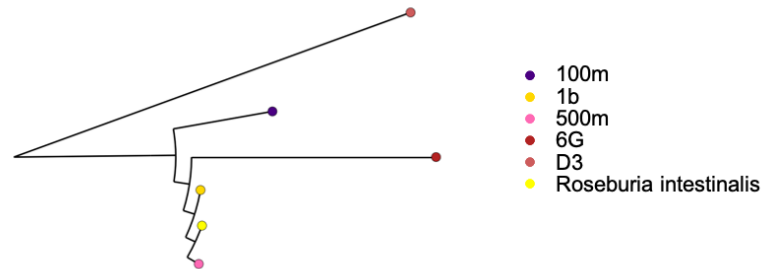

D

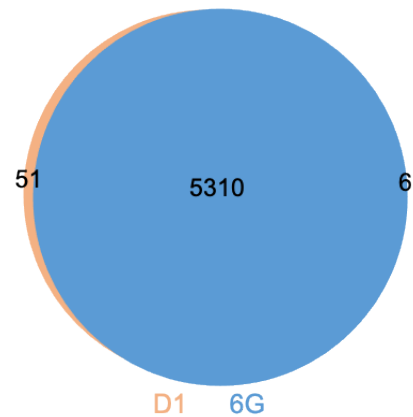

E

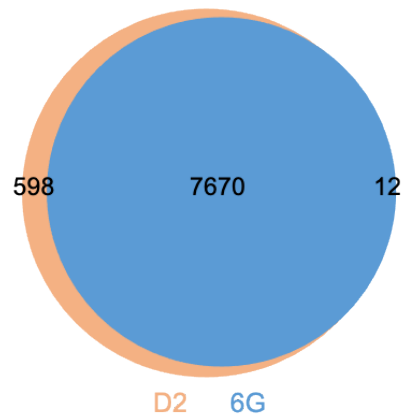

F

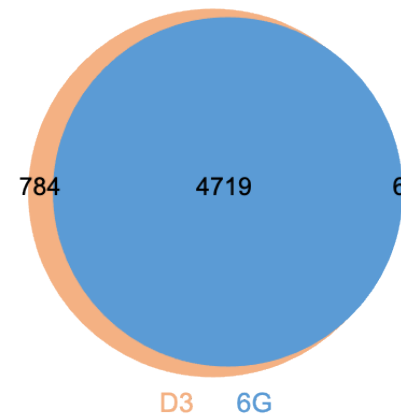

Supplement: Supplementary file 1 [file Data_Sheet_1.zip › supplementary/FigureS8.pdf]

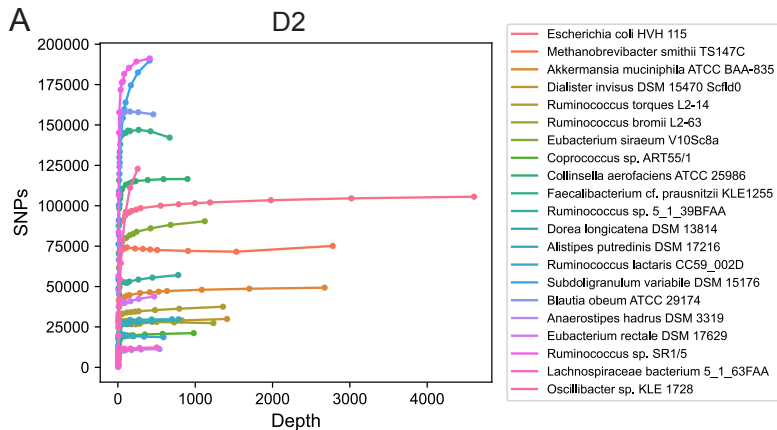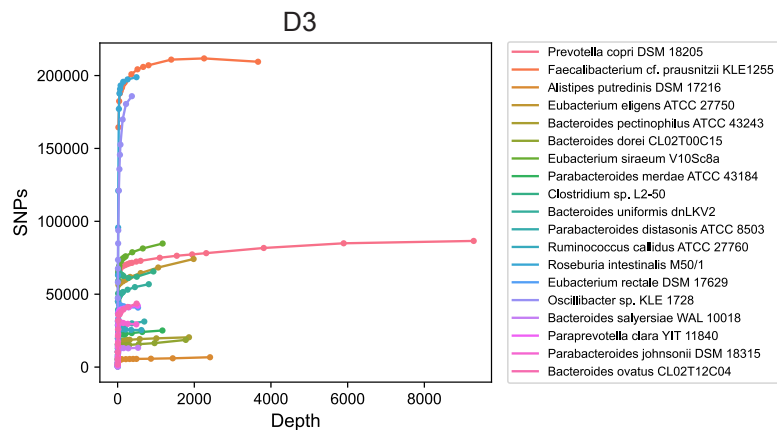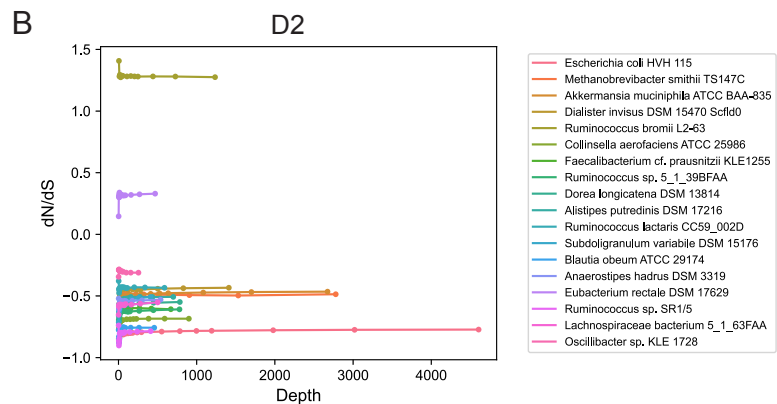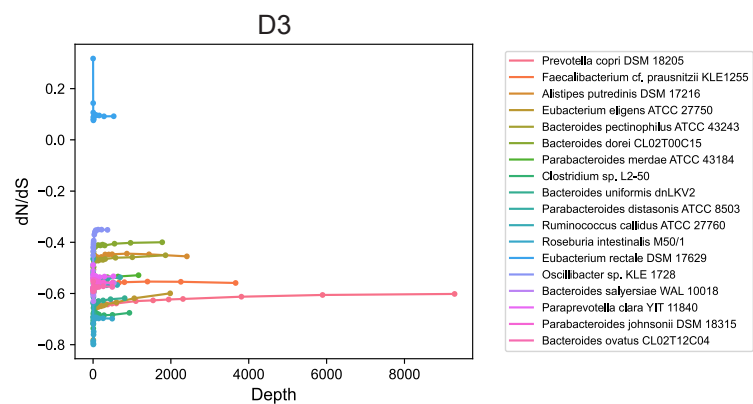

Supplement: Supplementary file 1 [file Data_Sheet_1.zip › supplementary/FigureS9.pdf]
